# Construction of prediction model of inflammation related genes in idiopathic pulmonary fibrosis and its correlation with immune microenvironment

**DOI:** 10.3389/fimmu.2022.1010345

**Published:** 2022-12-19

**Authors:** Ying-Qiu Yin, Feng Peng, Hui-Jing Situ, Jun-Ling Xie, Liming Tan, Jie Wei, Fang-fang Jiang, Shan-Qiang Zhang, Jun Liu

**Affiliations:** ^1^ Department of Respiratory Medicine, Yue Bei People’s Hospital, Shantou University Medical College, Shaoguan, Guangdong, China; ^2^ Department of Radiotherapy, Yue Bei People’s Hospital, Shantou University Medical College, Shaoguan, Guangdong, China; ^3^ Medical Research Center, Yue Bei People’s Hospital, Shantou University Medical College, Shaoguan, Guangdong, China

**Keywords:** idiopathic pulmonary fibrosis, inflammation, immune microenvironment, prognosis, ssGSEA

## Abstract

**Background:**

The role of inflammation in the formation of idiopathic pulmonary fibrosis (IPF) has gained a lot of attention recently. However, the involvement of genes related to inflammation and immune exchange environment status in the prognosis of IPF remains to be further clarified. The objective of this research is to establish a new model for the prediction of the overall survival (OS) rate of inflammation-related IPF.

**Methods:**

Gene Expression Omnibus (GEO) was employed to obtain the three expression microarrays of IPF, including two from alveolar lavage fluid cells and one from peripheral blood mononuclear cells. To construct the risk assessment model of inflammation-linked genes, least absolute shrinkage and selection operator (lasso), univariate cox and multivariate stepwise regression, and random forest method were used. The proportion of immune cell infiltration was evaluated by single sample Gene Set Enrichment Analysis (ssGSEA) algorithm.

**Results:**

The value of genes linked with inflammation in the prognosis of IPF was analyzed, and a four-genes risk model was constructed, including tpbg, Myc, ffar2, and CCL2. It was highlighted by Kaplan Meier (K-M) survival analysis that patients with high-risk scores had worse overall survival time in all training and validation sets, and univariate and multivariate analysis highlighted that it has the potential to act as an independent risk indicator for poor prognosis. ROC analysis showed that the prediction efficiency of 1-, 3-, and 5-year OS time in the training set reached 0.784, 0.835, and 0.921, respectively. Immune infiltration analysis showed that Myeloid-Derived Suppressor Cells (MDSC), macrophages, regulatory T cells, cd4+ t cells, neutrophils, and dendritic cells were more infiltrated in the high-risk group than in the low-risk group.

**Conclusion:**

Inflammation-related genes can be well used to evaluate the IPF prognosis and impart a new idea for the treatment and follow-up management of IPF patients.

## Introduction

Idiopathic pulmonary fibrosis (IPF) is one of the most frequent malignant diseases associated with interstitial pneumonia (1). IPF is chronic, progressive, and often occurs in men after the age of 50, but its specific pathogenesis remains to be studied further (2, 3). IPF is mainly caused by chronic inflammatory stimulation and pulmonary interstitial fibrosis proliferation, which often causes dyspnea in patients, and in severe cases, induces respiratory failure, leading to death (4). After the onset of IPF, its progress is relatively rapid, the prognosis is poor, and the average median survival time is < 4 years (5). Due to the deterioration of the environment and the increase in the aging population, the incidence rate of IPF also shows an increasing trend every year (6, 7). IPF has high heterogeneity, and there are great differences in the rate of disease progression between different patients (2). At present, although pirfenidone and nidanib have been approved for the clinical treatment of IPF, their clinical efficacy is still not ideal, and clinical studies show that they are unable to reduce mortality (8–10). Research shows that monitoring the disease process of IPF patients in order to implement precise and individualized management and supportive care helps improve the survival time and quality of life of IPF patients (11). However, effective prognostic indicators for IPF are still needed.

Transcriptomics has been paid increasing attention by researchers in the study of disease occurrence and development (12, 13). Presently with the advancement made in the field of genomics, a bulk of accessible genomic and clinical data is available, providing greater convenience and possibilities for the study of the occurrence of diseases. Recently, the biological processes and potential biomarkers related to the occurrence of diseases have been reported by many researchers through public databases (14, 15). However, the role of the genes related to inflammation in the prognosis of IPF needs to be studied in further detail. Compared with lung biopsy, bronchoalveolar lavage fluid (BALF) has better patient compliance and wider application prospects in the diagnosis of lung diseases (16, 17). In individuals with IPF in BALF samples, researchers observed a remarkable increase in the amount of platelet-derived growth factor (PDGF) (18). PDGF has been proved to be related to pulmonary angiogenesis and pulmonary hypertension and is significantly involved in the early stage of pulmonary fiber formation (19). In addition, TGF-β was also found to be significantly overexpressed in BALF of IPF patients (20). Studies have shown that TGF-β is expressed in alveolar epithelial cells and macrophages, and its overexpression can lead to pulmonary fibrosis in rats (21). These results suggest that BALF can be used to analyze the occurrence of pulmonary fibrosis and its development.

In this study, the mRNA expression microarray datasets of BALF and PBMC were obtained from Gene Expression Omnibus (GEO) database. This study investigates the involvement of genes linked with inflammation in the prognosis of IPF, screens and develops a risk model for the prognosis evaluation of IPF with the help of a variety of machine learning methods, and verifies its robustness. This research gives a new insight to understand the role that inflammation plays in the prognosis of IPF and develops a risk model of genes related to inflammation that can be employed for the prediction of the overall survival.

## Methods and materials

### Acquisition and processing of microarray data

The expression microarray data of bronchoalveolar lavage (BAL) cells (GSE70866), were obtained from the GEO database. Among them, there were 20 individuals from the control group and 112 individuals with IPF patients. A total of 132 mRNA microarray data were obtained from Agilent-028004 SurePrint G3 Human GE 8x60K Microarray, GPL14550 platform. The microarray data of 64 IPF patients was obtained through Agilent-039494 SurePrint G3 Human GE v2 8x60K Microarray, GPL17077 detection platform. In addition, the gene expression microarray data of peripheral blood mononuclear cell (PBMC), GSE28042, was also obtained from the GEO database. This dataset contains 75 patients with IPF, and their microarray information detection was done by Agilent-014850 Whole Human Genome Microarray 4x44K G4112F platform. The clinical data and OS follow-up information of all patients with IPF were also obtained from the GEO database. The “SVA” function of the “limma” package was used to correct the background, standardize and remove the batch effect of three groups of original data sets. Among them, 132 patients from the GSE70866 dataset GPL14550 were used as the discovery set, 64 IPF patients from the GPL17077 platform, and 75 IPF patients from the GSE28042 queue were used as the independent validation set.

### Acquisition and analysis of inflammation-related genes

The molecular signatures database (MSigDB, http://www.GSEa-msigdb.org/GSEa/index.jsp) was employed to obtain the inflammatory response-related gene set. To determine the differentially expressed inflammation-linked genes the “limma” package was utilized, and p < 0.05 and False Discovery Rate (FDR) < 0.05 were considered as cut-off values. The gene ontology (GO) and Kyoto Encyclopedia of Genes and Genomes (KEGG) were used to enrich the differentially expressed genes (DEGs) using the “clusterProfiler” package.

### Stratification of IPF patients

The identification of genes linked with the prognosis of IPF was made by Univariate Cox regression. The least absolute shrinkage and selection operator (Lasso) regression is often used to determine characteristic variables, which can well retain valuable variables and avoid overfitting. In this study, Lasso regression was employed for the identification of characteristic genes related to OS. Stochastic forest analysis is an analytical method that uses a decision tree to evaluate the importance of variables. The random forest was utilized to rank the involvement of genes in OS, and then the intersection of the genes identified by the two methods was taken. Finally, the gene set independently related to OS was identified through multivariate stepwise Cox regression, and a risk stratification model was constructed.

### Immune correlation analysis

Through the single sample Gene Set Enrichment Analysis (ssGSEA) algorithm of the “GSVA” R package, the level of immune cell infiltration was evaluated in the discovery set and validation set, respectively, in accordance with the gene expression array. CIBERSORT algorithm (https://cibersortx.stanford.edu/) was also used to evaluate the immune cell infiltration.

### Bioinformatics analysis and statistical analysis

R software was employed for the complete data analysis of this study. The “ggplot2” package was used to perform chart drawing. “Survival” and “Surviviner” packages were used to draw Kaplan Meier (K-M) curves, and for the evaluation of the statistical significance of survival time distribution, the log-rank test was employed. The univariate and multivariate analysis was performed using the “survival” package. Wilcox test was employed to evaluate the variation between non-normal distribution variables, and the value of p < 0.05 was deemed statistically significant. The receiver operating characteristic (ROC) curve was drawn employing the ‘survivalROC’ package, and the area under the curve (AUC) was also calculated using the package. The “RMS” package was employed to draw the calibration of nomograms. Pearson correlation analysis was performed to evaluate the relationship between immune cell proportion and risk score.

## Results

### Differential analysis of inflammation-related genes

**Figure 1:**
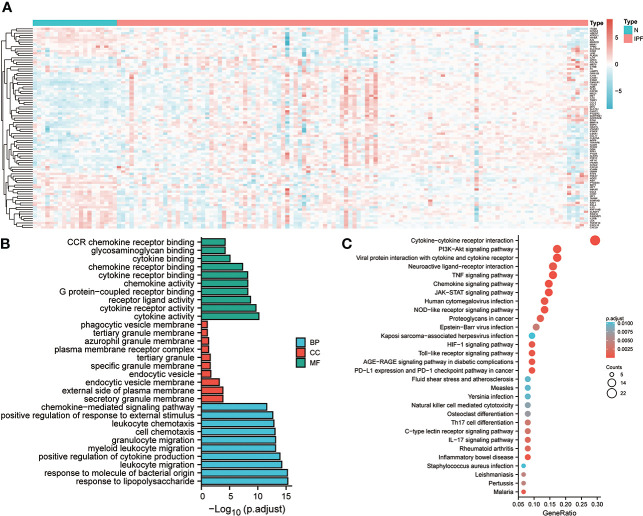
Expression and enrichment analysis of inflammation-linked genes in IPF. **(A)** Heat map of the expression of genes linked with inflammation in the discovery set. **(B)** GO enrichment analysis of DEGs. **(C)** KEGG analysis of DEGs.

In the alveolar lavage fluid of 20 healthy controls and 112 individuals with IPF, a total number of 90 differentially expressing inflammation-related genes were identified, and their expression in the two groups is shown in the heat map (**Figure 1A**). Subsequently, Gene Ontology (GO) enrichment analysis revealed that the Biological Process (BP) to which these 90 differential genes were mainly enriched in response to lipopolysaccharide, leucocyte migration, and positive regulation of cytokine production. The enriched Cellular Component (CC) was the granular secretory membrane, the external side of the plasma membrane, and the endocytic vesicle membrane. The Molecular Function (MF) enriched were mainly cytokine activity, cytokine receptor activity, and receptor-ligand activity (**Figure 1B**). These DEGs were mainly linked to the PI3K-Akt signaling pathway, Cytokine-cytokine receptor interaction, neuroactive ligand-receptor interaction, TNF signaling, and other pathways, as shown by KEGG enrichment analysis. (**Figure 1C**).

### Construction of inflammation-related gene model

**Figure 2:**
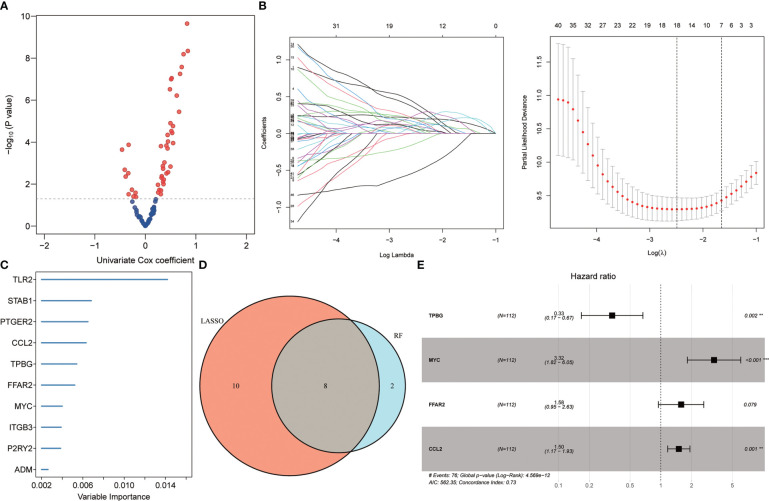
Identifying prognostic biomarkers. **(A)** Univariate Cox regression identifying genes associated with IPF prognosis. **(B)** Lasso regression further identifying prognosis-related genes. **(C)** Random Forest identifying the top 10 genes that are relatively important in prognosis. **(D)** Wayne diagram identifying the common genes selected by the two methods. **(E)** Multivariate stepwise Cox regression finally determined the prognosis-related genes.

Initially, the identification of 46 genes associated with the poor prognosis of individuals with IPF from 90 DEGs was carried out by univariate Cox regression, including 10 negative correlations and 36 positive correlations (**Figure 2A**). Subsequently, 18 of the most characteristic inflammation-related genes were identified from 46 prognosis-related genes by Lasso regression (**Figure 2B**). Afterward, the prognostic genes were sorted, and the top 10 genes of relative importance were identified based on the random forest algorithm (**Figure 2C**). In order to identify more robust prognostic markers, the intersection of genes was taken from Lasso regression and random forest screening, and 8 genes were obtained in total (**Figure 2D**). Further, a 4-gene model related to inflammation was constructed through multivariate stepwise Cox regression (**Figure 2E**). Its risk score (RS) =-1.096* expression of TPBG + 1.199* expression of MYC + 0.457* expression of FFAR2 + 0.405* expression of CCL2.

### Prognostic importance of the four-gene risk model

**Figure 3:**
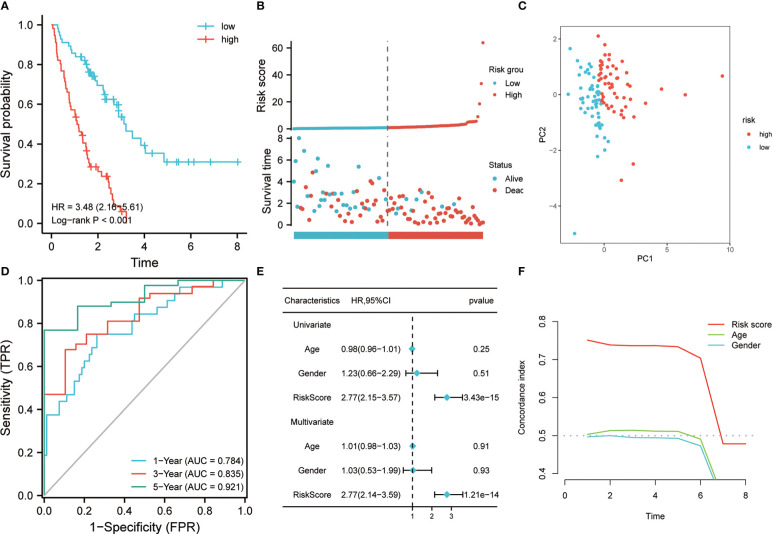
Prognostic value of risk model in the training cohort. **(A)** K-M method was used to draw the survival curve according to risk score, and for comparison, a log-rank test was employed. **(B)** The distribution of risk score and survival status between high and low-risk groups. **(C)** To evaluate the differentiation between groups with high and low risk, PCA was utilized. **(D)** Through ROC analysis, the predictive effect of the risk model in the training queue was evaluated. **(E)** Univariate and multivariate Cox analysis. **(F)** C-index analysis was used to evaluate the prediction ability of the model.

A prognostic classifier was constructed in the discovery set based on this four-gene risk model. As per the median value of its RS, the discovery set was sorted into a low-risk group and a high-risk group. The K-M curve revealed that the individuals in the high-risk group had a worse OS time as compared to the low-risk patients’ group (hazard ratio (HR) = 3.48, p <0.001, **Figure 3A**). Remarkable high scores were shown by patients in the high-risk group, and higher mortality was also seen in this group (**Figure 3B**). PCA analysis revealed that there was a notable distribution variation in the low-risk group and the high-risk group (**Figure 3C**). Then, ROC analysis was utilized to evaluate the prediction efficiency of the risk model. It was shown by the results that 1-year, 3-year, and 5-year survival AUC values predicted by the model were 0.784, 0.835, and 0.921, respectively, showing a strong predictive ability (**Figure 3D**). Univariate and multivariate Cox analysis revealed that our risk model worked as an independent risk factor for the poor prognosis of individuals with IPF (**Figure 3E**). The concordance index showed that our model is much better than the prediction efficiency of age and gender, which has been maintained above 0.7 (**Figure 3F**). To illustrate the superiority of the 4-gene model, we compared it with the other 4 genes that were common to LASSO and RF but not included in the model. The K-M curve revealed significant survival differences between high-risk and low-risk groups (HR=3.3, P<0.001) (**Figure S1A**). The ROC analysis indicated that the risk model also has certain prediction ability in predicting 1- and 3- years, with an AUC of 0.700 and 0.756 (**Figure S1B**). This shows that the model constructed based on the multivariate Cox stepwise regression method is significantly superior to the model selected by random variables. In addition, we also compared our model with LASSO-based model and RF-based model. ROC analysis showed that the 4-gene signature exhibited a higher power of risk prediction than the 7-gene model based on LASSO regression (**Figures S2A, B**). Although, ROC analysis showed that the 10-gene signature (RF-based model) exhibited a higher power of risk prediction than the 4-gene model (**Figures S2C, D**). However, the number of genes in the 4-gene model is much less than the number of 10-gene model. In addition, we also performed multivariate Cox stepwise regression on these 10 genes, and finally the 4-gene model still had the lowest Akaike information criterion (AIC).

### Verification of risk model

**Figure 4:**
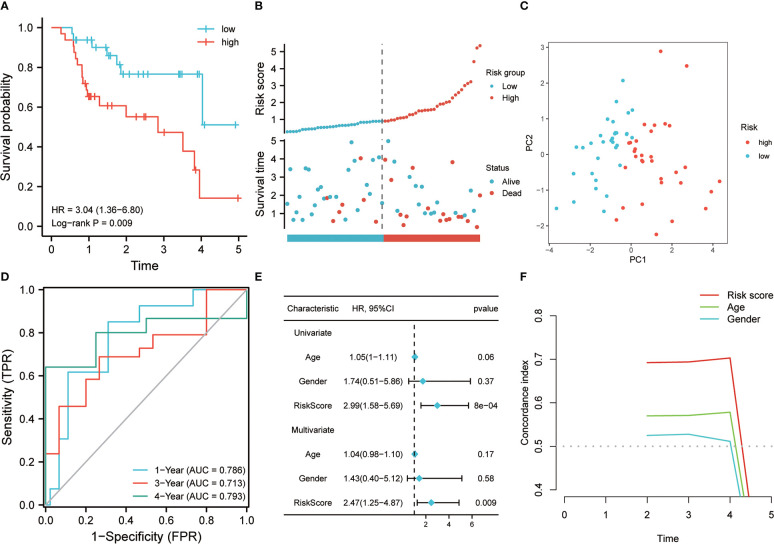
The effectiveness of the risk model was verified in the validation set. **(A)** K-M method was used to draw the survival curve according to risk score, and for the comparison, the log-rank test was employed. **(B)** The distribution of risk score and survival status between high and low-risk groups. **(C)** To evaluate the differentiation between groups with high and low risk, PCA was utilized. **(D)** Through ROC analysis, the predictive role of the risk model in the validation queue was evaluated. **(E)** Univariate and multivariate Cox analysis. **(F)** C-index analysis was used to evaluate the prediction ability of the model.

**Figure 5:**
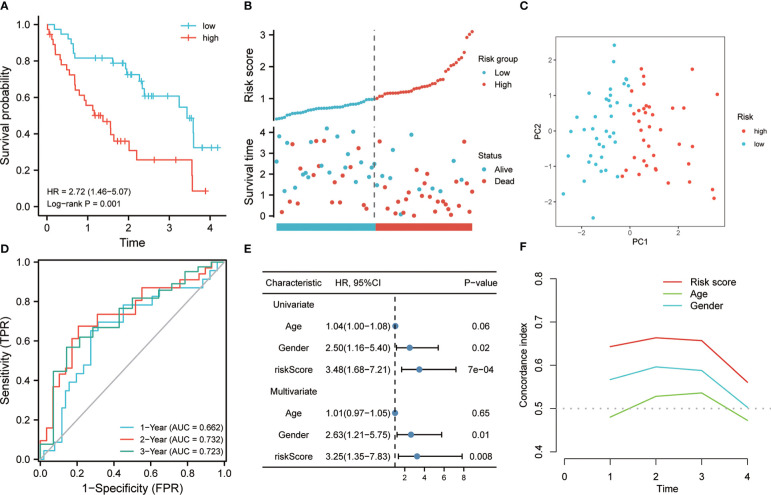
The effectiveness of the risk model was verified in the validation set. **(A)** K-M method was used to draw the survival curve based on risk score, and for comparison, a log-rank test was employed. **(B)** The distribution of RS and survival status between high and low-risk groups. **(C)** To evaluate the differentiation between groups with high and low-risk, PCA was utilized. **(D)** Through ROC analysis, the predictive role of the risk model in the validation cohort was evaluated. **(E)** Univariate and multivariate Cox analysis. **(F)** for the evaluation of the prediction ability of the model, C-index analysis was employed.

**Figure 6:**
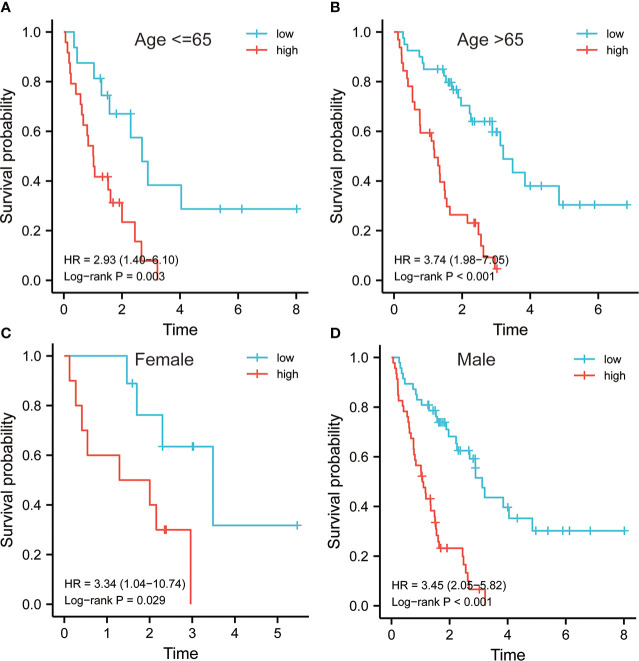
Survival analysis of risk model in different subgroups. Survival analysis of risk models in the subgroup of age ≤ 65 **(A)** and greater than 65 **(B)**. Survival analysis of risk models in female **(C)** and male **(D)**.

The 64 patients from the GSE70866 dataset GPL17077 platform were used as the validation set. K-M analysis revealed that the RS showed a certain correlation with the OS time, and the low RS predicted a better prognosis (**Figure 4A**). The distribution of RSs shows that the group with high risk has higher scores and higher mortality (**Figure 4B**). PCA analysis highlighted that the groups with high and low risk exhibited remarkably different clustering trends (**Figure 4C**). ROC analysis revealed that good prediction efficiency in the validation set was shown by the model, and its respective AUC values in 1, 3, and 4 years were 0.786, 0.713, and 0.793(**Figure 4D**). Univariate and multivariate Cox analysis also highlighted that this risk model still worked as an independent risk factor for the poor prognosis of individuals with IPF in the validation set (**Figure 4E**). C-index also showed the strong prediction ability of the model (**Figure 4F**). Considering that most of the BALF were immune cells, the predictive value of this model was verified further in the RNA-seq of PBMC of 75 IPF patients from the GSE28042 cohort. It was revealed by the K-M curve that the group with high risk in the GSE28042 cohort still showed a poor OS time (**Figure 5A**). The distribution of RSs and the correlation between RSs and survival data showed that high-risk groups had higher RSs and higher mortality (**Figure 5B**). As per the risk model and genome expression, PCA proved that the distribution in the group with low risk and group with high risk highlighted notable cluster variations (**Figure 5C**). It was also shown by ROC analysis that in the external validation set, the risk model showed good prediction efficiency, with respective AUC of 0.662, 0.732, and 0.723 in 1, 2, and 3 years (**Figure 5D**). Univariate and multivariate Cox regression analysis showed that for the poor prognosis of IPF in the external validation set, the risk model still worked as an independent risk factor (**Figure 5E**). Similarly, C-index analysis also indicated that the risk model performed well in predicting the OS time of 1, 2, and 3 years (**Figure 5F**). Further, we analyzed the prognostic significance of this risk model in different ages and gender. The results revealed that the high RS was still linked with poor OS time in people younger than 65 years old or older (**Figures 6A, B**). Similarly, a higher RS in both men and women predicts a poor prognosis (**Figures 6C, D**). These results indicate that our risk model has strong predictive power in the prognosis evaluation of IPF patients.

### Comprehensive analysis of immune cell infiltration

**Figure 7:**
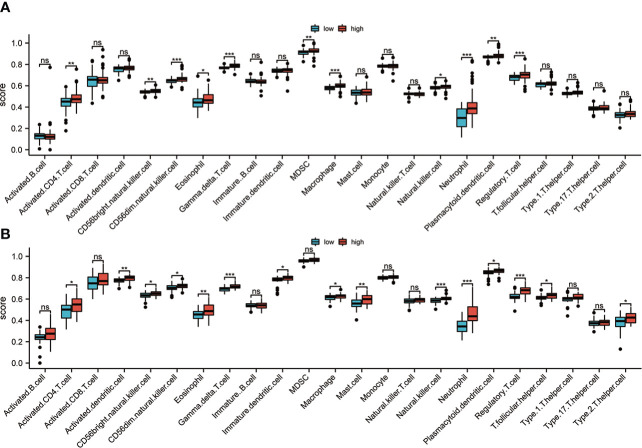
Immune infiltration analysis. **(A)** In the discovery set, the immune cell infiltration between groups with high and low risk was evaluated based on the ssGSEA algorithm. **(B)** In the validation set, the immune cell infiltration between high-risk and low-risk groups was analyzed based on the ssGSEA algorithm ns, non significance; *p<0.05; **p<0.01; ***p<0.001.

As we know, BAL is rich in immune cells, so the ssGSEA algorithm was used for the evaluation of the variations in the distribution of immune cells in low- and high-risk groups. As expected, the results in the discovery set showed higher infiltration of macrophage, neutrophil, regulatory T cell, and NK cell infiltration in the high-risk group (**Figure 7A**). Further, the distribution of immune cells in the validation cohort from PBMC was also analyzed, and the results were also consistent with the discovery set (**Figure 7B**). The group with high risk had more macrophages, neutrophils, regulatory T, and NK cells among others. The similar results were confirmed by Pearson correlation analysis (**Figures S3A, B**). In addition, CIBERSORT algorithm also showed that the risk score was positively correlated with Neutrophils, T cells gamma, T cells memory activated, and T cells CD8 (**Figure S3C, D**). These results suggest that there is higher infiltration of inflammatory cells in the group with high risk, and the stimulation of these inflammatory cells are possibly linked with the poor prognosis of IPF.

### Construction of nomogram

**Figure 8:**
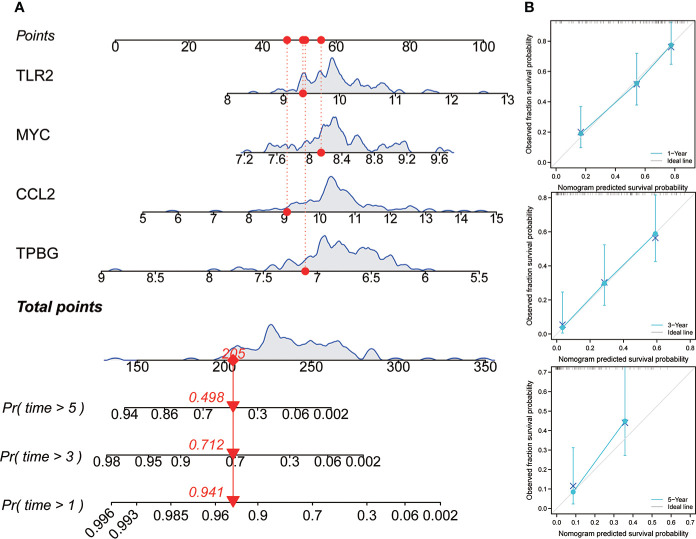
Construction and evaluation of nomogram. **(A)** Nomogram was constructed based on the expression values of four genes to predict the OS rate at 1, 3, and 5 years. **(B)** The calibration curve was used to assess the nomogram.

In order to promote the application of this risk model in clinical practice and facilitate the operability of clinical workers, a four-gene nomogram was constructed (**Figure 8A**). The nomogram can be used to assess the 1-, 3-, and 5-year OS rates of individuals with IPF. In addition, to find out the nomogram’s effectiveness, a calibration chart was drawn, and the results revealed that the nomogram had a very good prediction performance, which was almost close to the ideal model in the prediction of 1 and 3 years (**Figure 8B**).

## Discussion

Although the specific pathogenesis of IPF is still controversial, it has been reported by many studies that chronic inflammatory stimulation is crucially involved in the development and progression of IPF (22, 23). At present, researchers generally believe that chronic inflammation caused by lung epithelial cell injury is repeatedly stimulating, resulting in excessive wound repair and tissue cell remodeling, and fibrosis (24, 25). Fibroblasts can induce the secretion of interleukin-33, IL-13, IL-6, IL-1, PDGF, fibroblast growth factor, and TGF-B, and promote their differentiation into myofibroblasts (5, 26). In addition, the disturbance of the homeostasis of various immune cells is also considered to be an important reason for the formation of lung fibers (27). Macrophages are considered to be the main culprit of balance disorder in the process of wound healing (28, 29). Macrophages can also further promote the process of fibrosis by secreting related cytokines (26). However, the underlying mechanism of inflammation in the formation of pulmonary fibrosis still needs further exploration. In this study, we evaluated the expression of inflammation-linked genes in patients with IPF through the microarray expression chip of BALF and explored the link between their expression and the prognosis of patients. GO and KEGG enrichment analysis showed that these DEGs were also involved in a variety of viral infection related pathways, including viral protein interaction with cytokine and cytokine receptor, Human cytomegalovirus infection, and Epstein-Barr virus infection. This is also consistent with recent studies showing that the presence of viral infections is a significant risk factor for IPF pathogenesis (30, 31). In addition, a new four-gene risk model was designed based on multiple machine learning. The robustness of the model was verified in two independent validation queues.

Inflammation and immune microenvironment changes are the basis of IPF. Regulatory T cells are crucial in maintaining the stability of the immune microenvironment (32). Studies showed that regulatory T cells (Tregs) were significantly enriched in the lungs of IPF mice, and they were also found to be significantly activated in the peripheral blood of individuals with IPF, which was positively correlated with the progress of the disease (33). Tregs can also promote the progress of IPF by inducing the activation of Th2 and Th17 cells (34). Studies have shown that Th2 and Th17 cells can promote the disease process of pulmonary fibrosis, and their increase is associated with a positive correlation with the severity of the disease (35, 36). The researchers’ knowledge regarding the role that macrophages have in the development of IPF is also constantly refreshed. Recently, results from single-cell RNA sequencing have identified many new macrophage subpopulations, and their different subpopulations are specifically expressed in different stages of IPF development (37). Myeloid-derived suppressor cells (MDSCs) are also an important part of regulating the immune microenvironment. Studies have shown that MDSCs are found to increase significantly in the lungs, and peripheral blood of IPF patients and are positively correlated with poor lung function (38, 39). These findings are compatible with the findings of this investigation, according to several studies. The results of ssGSEA analysis in this study showed that in the group with high risk, there was enhanced infiltration of MDSC, macrophages, and regulatory T cells, which also predicted a worse OS time. In addition, we also found that in the high-risk group, there was higher infiltration of CD4, T cells, NK cells, dendritic cells, and neutrophils, which may further increase the inflammatory response and aggravate the disease process. A new perspective may be provided by our results for further understanding the characteristics of immune cell infiltration in IPF.

A number of studies have described the prognostic value of gene signature in IPF. Recently, Casanova NG et al. proposed a 21-gene prognostic model to predict the overall survival for IPF patients (40). Li X et al. developed a 9-gene prognostic signature based on hypoxia-immune-related genes (41). In this study, four inflammation-related genes, trophoblast glycoprotein (tpBG), Myc, free fatty acid receptor 2 (ffar2), and CCL2, were identified as important prognostic markers. It is worth mentioning that the 4-gene signature has fewer genes, but exhibits greater predictive power. TPBG belongs to carcinoembryonic antigen and is mainly involved in cell adhesion (42). It is expressed highly in numerous tumor cells and is related to the poor prognosis of cancer (43, 44). However, there are few reports about its role in IPF. In this study, our results show that TPBG can be used as a good prognostic indicator of IPF. Reportedly, as a proto-oncogene, MYC has a crucial role in cell cycle progression, apoptosis, and cell transformation (45). Research has shown that MYC is up-regulated in pulmonary fibrosis cells of IPF mice and promote the growth and differentiation of pulmonary fibrosis cells by regulating the transcription of miR-9-5p (46). Yin X et al. also reported that MYC could promote the accumulation of HK2 in human and mouse lung fibroblasts, thereby increasing the proliferation and migration of fibroblasts (47). The protein encoded by FFAR2 is a member of the GP40 family that mainly play a role in the regulation of inflammatory response and plasma lipid level (48). Maslowski KM et al. confirmed that short chain fatty acids promote the occurrence of the inflammatory response by binding to FFAR2 (49). The results of Sencio V et al. showed that FFAR2 was also related to the secondary infection of pneumococcus after influenza infection (50). However, we have little knowledge about the role of FFAR2 in IPF. A new direction may be provided by our results for understanding the occurrence and progress of IPF. There is increasing evidence that CCL2 is linked with the onset of pulmonary inflammatory diseases. Several studies have suggested that the concentration of CCL2 in BALF of individuals with IPF has increased significantly, and a significant correlation exists between its expression level and the increased risk of death (51). In addition, CCL2 can also combine with related transcription factors to regulate the proliferation of fibroblasts and promote the differentiation of fibroblasts. Our results further prove that CCL2 from BALF can be used as an effective predictor of the poor prognosis of IPF.

While this study has many strengths, it has some limitations as well. This study analyzed whole of IPF patients, and did not distinguish between rapidly progressive type and slowly progressive type. In addition, this remains to be verified by either further functional studies *in vitro* or *in vivo*.

## Conclusion

In conclusion, this report reveals the importance of genes linked with inflammation in the prognosis of individuals with IPF. A new four-gene risk model based on BALF expression profile was built to predict the progress and prognosis of IPF, which is conducive to further monitoring and management of IPF patients.

## Data availability statement

The original contributions presented in the study are included in the article/**Supplementary Material**. Further inquiries can be directed to the corresponding authors.

## Author contributions

JL, S-QZ, Y-QY, and FP all participated in research design and drafting the manuscript. JL, S-QZ, Y-QY, FP, H-JS, J-LX, LT, JW, and F-FJ took part in the data collection process of the study and is responsible for the content of the manuscript. All authors read and approved the final manuscript.

## Supplementary material

Supplementary Figure 1 Prognostic value of risk model. **(A)** K-M method was used to draw the survival curve based on risk score, and for comparison, a log-rank test was employed. **(B)** Through ROC analysis, the predictive role of the risk model was evaluated.

Supplementary Figure 2 Prognostic value of risk model. **(A)** K-M method was used to draw the survival curve based on risk score in LASSO-based model. **(B)** ROC analysis was used to evaluate the predictive power of LASSO-based model. **(C)** K-M method was used to draw the survival curve based on risk score in RF-based model. **(D)** ROC analysis was used to evaluate the predictive power of RF-based model.

Supplementary Figure 3 Correlation analysis of risk score and immune cell. Pearson correlation analysis between risk score and immune cell infiltration abundance based on ssGSEA **(A)** and CIBERSORT **(C)** methods in discovery set. Pearson correlation analysis between risk score and immune cell infiltration abundance based on ssGSEA **(B)** and CIBERSORT **(D)** methods in validation set.
