# Supplementary figures and images for: Construction of prediction model of inflammation related genes in idiopathic pulmonary fibrosis and its correlation with immune microenvironment
Source: Front Immunol. 2022 Dec 19;13:1010345. doi: 10.3389/fimmu.2022.1010345 (PMC9806212; doi:10.3389/fimmu.2022.1010345)

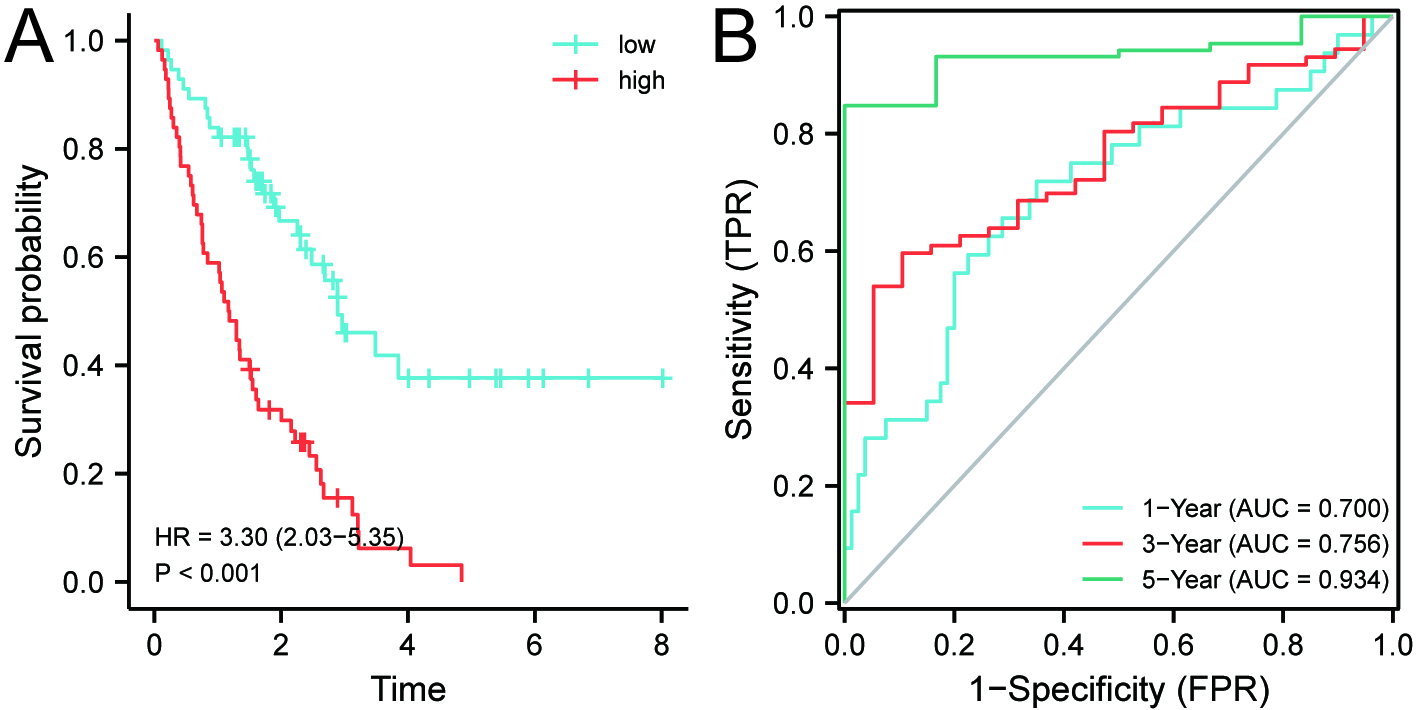

Supplement: Supplementary Figure 1 — Prognostic value of risk model. (A) K-M method was used to draw the survival curve based on risk score, and for comparison, a log-rank test was employed. (B) Through ROC analysis, the predictive role of the risk model was evaluated. [file Image_1.tif]

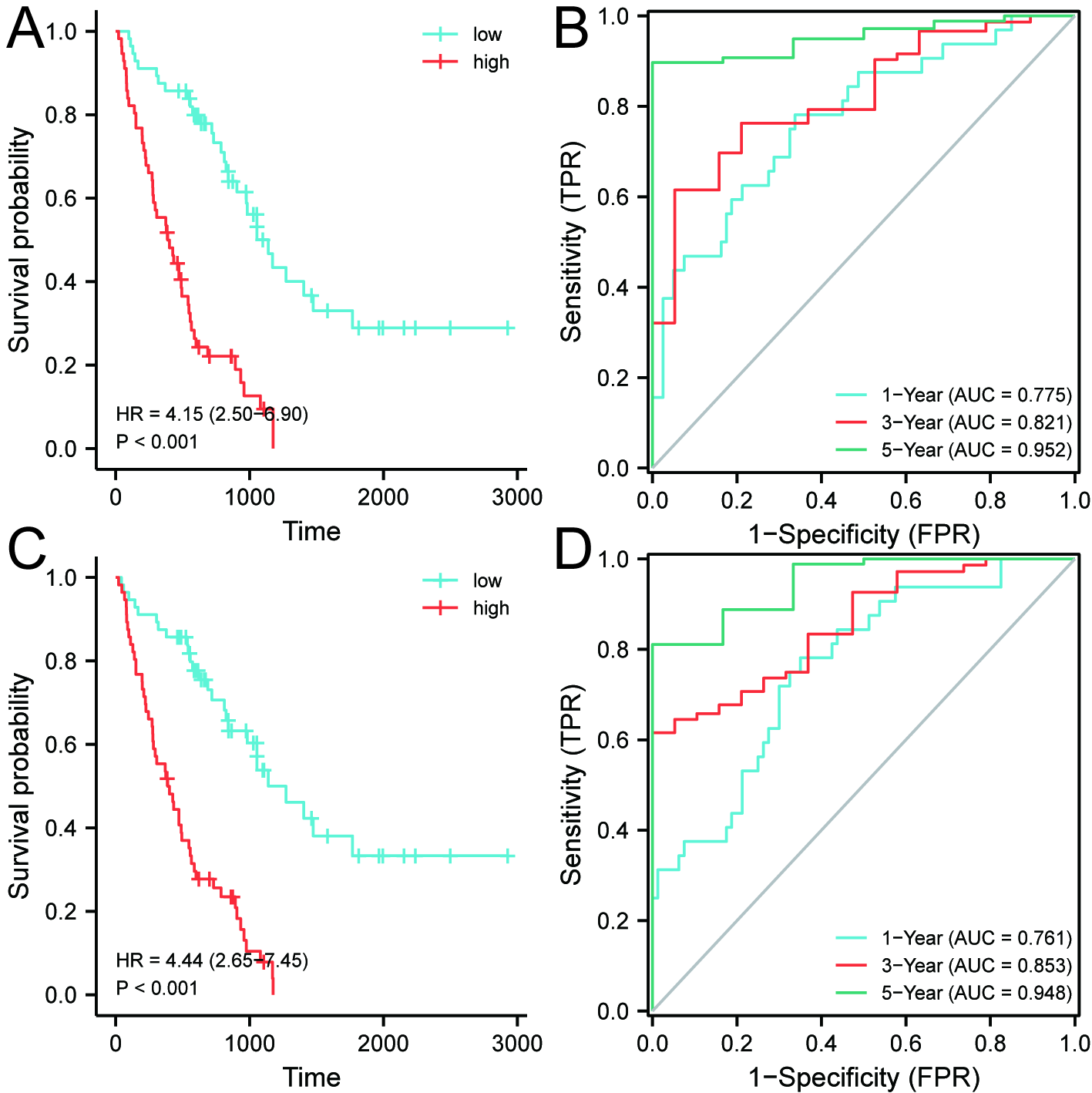

Supplement: Supplementary Figure 2 — Prognostic value of risk model. (A) K-M method was used to draw the survival curve based on risk score in LASSO-based model. (B) ROC analysis was used to evaluate the predictive power of LASSO-based model. (C) K-M method was used to draw the survival curve based on risk score in RF-based model. (D) ROC analysis was used to evaluate the predictive power of RF-based model. [file Image_2.tif]

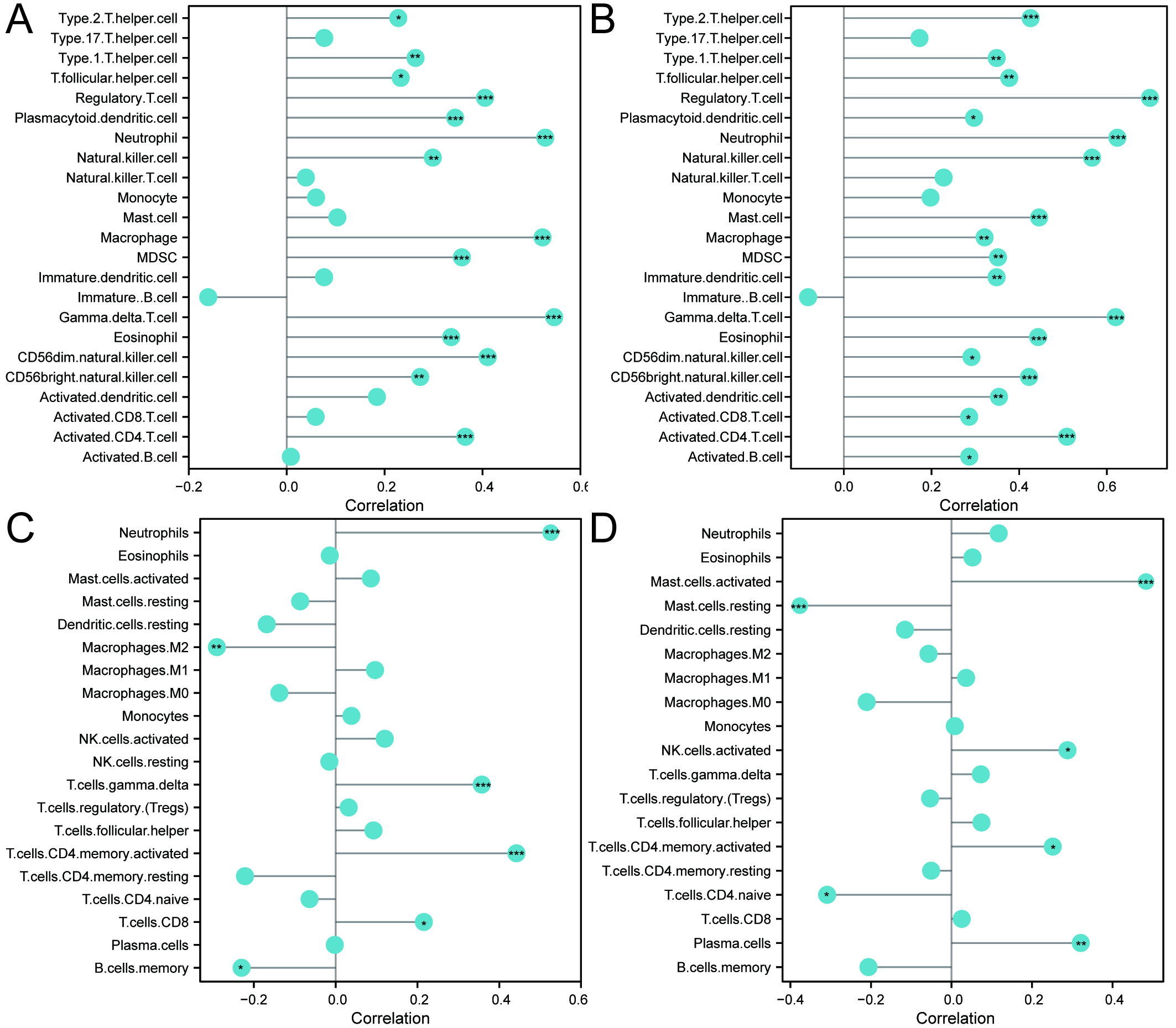

Supplement: Supplementary Figure 3 — Correlation analysis of risk score and immune cell. Pearson correlation analysis between risk score and immune cell infiltration abundance based on ssGSEA (A) and CIBERSORT (C) methods in discovery set. Pearson correlation analysis between risk score and immune cell infiltration abundance based on ssGSEA (B) and CIBERSORT (D) methods in validation set. [file Image_3.tif]
